# Optimizing Nutritional Support Through the Retromolar Space

**DOI:** 10.7759/cureus.83173

**Published:** 2025-04-29

**Authors:** Shikha Yadav, Yugesh Chandra, Navya Gella, Prashanthi Polakonda, Anuvindha JS

**Affiliations:** 1 Department of Dentistry, All India Institute of Medical Sciences, Mangalagiri, Mangalagiri, IND; 2 Department of Oral and Maxillofacial Surgery, All India Institute of Medical Sciences, Mangalagiri, Mangalagiri, IND

**Keywords:** buccal corridor, diet plan, imf, maxillofacial trauma, maxillomandibular fixation (mmf), nutritional intervention, quality of life (qol), retromolar space (rms), weight loss

## Abstract

Maxillofacial trauma resulting from injuries or surgical procedures significantly impacts patient well-being and recovery outcomes. Adequate nutrition is pivotal in wound healing and overall health during the post-injury phase. This technical report explores the critical relationship between nutrition and maxillofacial trauma management. We discuss the challenges maxillomandibular fixation poses and propose to address nutritional needs effectively through the retromolar space.

## Introduction

The relationship between nutrition and wound healing post-injury or surgery has been recognized for centuries, emphasizing its critical role in patient recovery. Malnutrition or inadequate nutrient intake significantly affects outcomes, increasing complication rates and healthcare costs [1]. In the context of maxillofacial trauma management, clinicians face choices between open reduction and internal fixation (ORIF) and conservative methods such as maxillomandibular fixation (MMF). MMF achieved through techniques such as arch bars, Ernst ligatures, or bone-supported devices temporarily immobilizes the maxilla and mandible. However, this immobilization impacts nutritional intake and quality of life (QoL), especially after orthognathic surgery or when managing maxillary mandibular fractures [2]. Addressing prolonged MMF-induced malnutrition is imperative for enhancing patient well-being and optimizing recovery outcomes. Regardless of the choice between MMF with or without ORIF, the functional specificity of the oral cavity may lead to a reduction in physical strength (weight, muscle mass, grip strength, etc.). A team-based approach toward tailored nutrition is essential [3-5].

## Technical report

**Figure 1:**
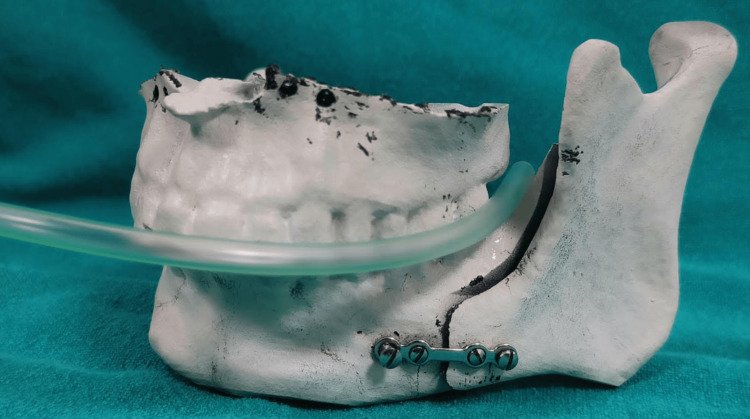
Feeding tube entering via the buccal corridor into the retromolar space.

In managing patients with MMF, maintaining oral hygiene and patient comfort can be challenging due to the constraints of a liquid diet. To mitigate these issues, we propose using a suction tube (e.g., F14) inserted through the buccal corridor into the posterior oral cavity via the retromolar space (RMS). This method facilitates the administration of semi-solid/liquid diets (millet/fruit smoothies, lentil/vegetable/chicken soups, milk/water, etc.), ensuring adequate nutritional intake during recovery (Figure 1).

**Figure 2:**
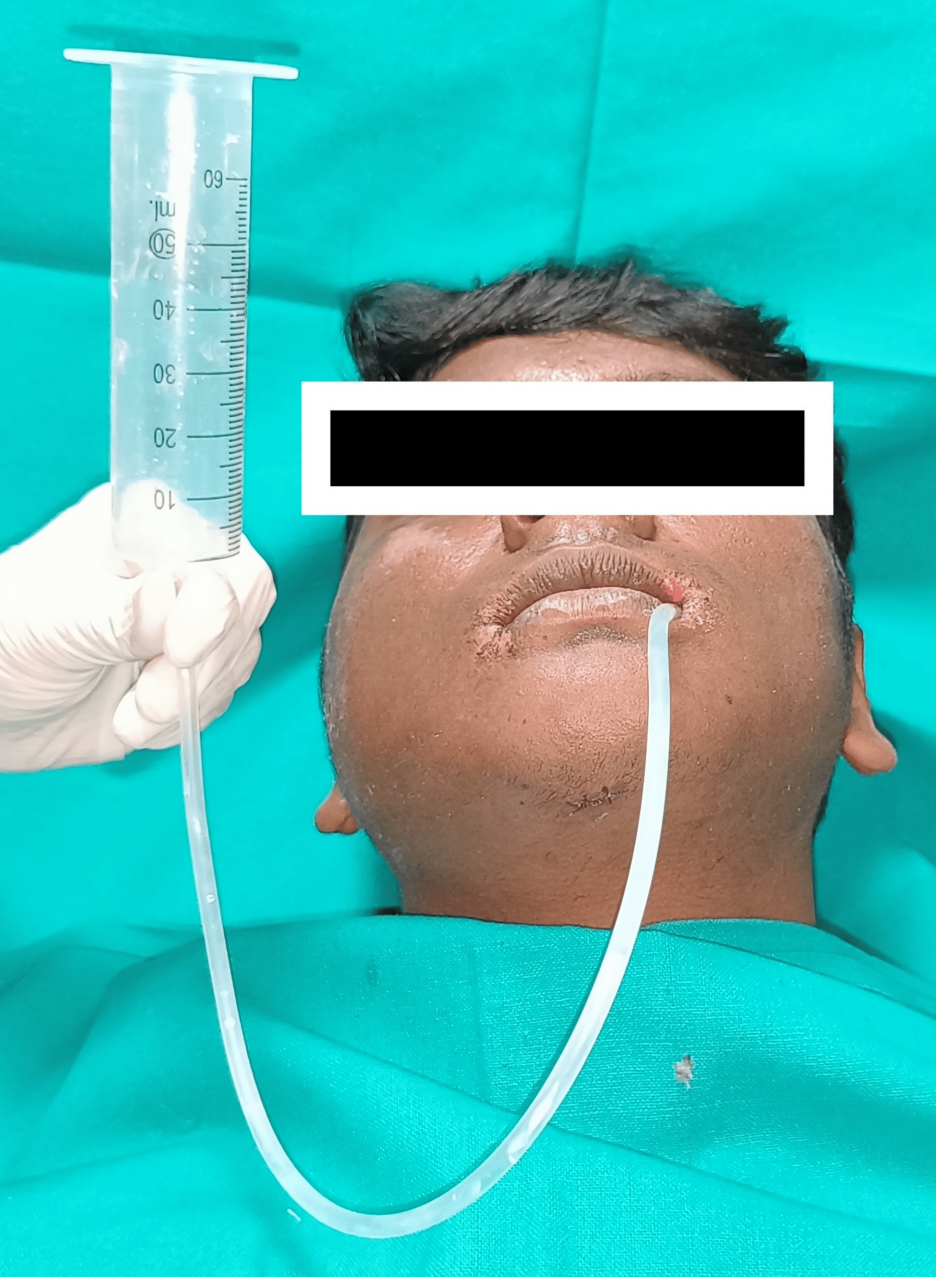
Feeding tube with a 50 mL syringe used to feed the patient.

The proposed technique offers several advantages, including controlled nutrition intake, ease of execution, and maintenance of surgical site hygiene. Compared to direct consumption using spoons/straws, tube feeding provides satiation, ease of administration, and cleanliness without the drawbacks of nasogastric feeding. The rate of administration can be easily controlled by pinching the tube. Post-meal tube cleaning can be easily accomplished by flushing it with water and air drying. The armamentarium, which is economically priced, can be conveniently replaced after a few uses. The learning curve for the patients and caretakers is smooth and short. This cost-effective method has high patient acceptance rates, making it a practical solution for feeding patients with MMF (Figure 2).

## Discussion

The RMS is anatomically bounded superiorly by the maxillary tuberosity and the area posterior to it; inferiorly by the retromolar trigone region; anteriorly by the most posteriorly erupted molar teeth; posteriorly by the anterior border of the ascending ramus of the mandible; medially by the lateral surfaces of the maxillary tuberosity, the last erupted molars, and the adjacent oral cavity; and laterally by the medial surface of the ascending ramus and the buccal vestibule [5]. RMS has been successfully utilized for tracheal tube placement and airway adjunct in adult and pediatric patients undergoing maxillofacial surgeries [6,7]. Despite the potential limitation of a fully erupted maxillary third molar, our experience suggests that the small size of the suction tube negates the need for tooth extraction. In case of paucity of the RMS, the surgeon can take a call concerning third molar tooth extraction.

Significant weight changes during MMF serve as key indicators of patient nutritional status, with studies indicating clinically substantial weight loss [8]. Malnutrition during MMF may necessitate nutritional supplements, underscoring the importance of systematic nutrition support during surgical and conservative treatments [4]. Manifestations of malnutrition include weight loss, neurological changes, skin alterations, and decreased serum proteins and lipids. The resurgence of MMF as a primary treatment during the COVID-19 pandemic, aimed at avoiding aerosol generation, further highlights this need. While nasogastric tube feeding can meet dietary requirements, its prolonged use is discouraged due to associated discomforts such as nausea, vomiting, aspiration pneumonia, ulceration, and faulty tube insertion.

Alternative methods, such as wafer placement between arches, are deemed cumbersome due to the need for fabricating a thicker splint. Moreover, these methods are not required in all cases post-orthognathic surgery and are never needed in maxillofacial trauma/pathology management [9]. A study by Ishikawa et al. demonstrated decreased complications and improved quality of the perioperative period for patients, attributed to the speed of administration and consistency of diet [10]. Consuming food directly using a spoon or straw can accumulate food particles around the teeth, orthodontic appliances, and surgical wounds in areas such as the retromolar and sulcus regions, potentially compromising oral hygiene. Moreover, eating with the jaws wired together can be a challenging experience, adding further difficulties for some patients.

## Conclusions

Optimal nutrition is crucial for expedited recovery, emphasizing the importance of meticulous nutritional planning in maxillofacial treatment. Even brief periods of malnutrition can significantly negatively affect wound healing. Hence, dietary deficiencies must be recognized early and repletion initiated as soon as possible. Nutrition significantly impacts feeding duration, comfort, patient satisfaction, and QoL, ultimately influencing wound healing and early discharge.
